# Joint association of sleep duration and physical activity with cognitive performance among Chinese adults: an analysis of nationally representative survey data

**DOI:** 10.3389/fpubh.2023.1244407

**Published:** 2023-11-03

**Authors:** Huan Tao, Tao Wang, Yong-Qian Jia

**Affiliations:** Department of Hematology, West China Hospital of Sichuan University, Chengdu, China

**Keywords:** sleep duration, physical activity, cognitive performance, joint association, nationally data

## Abstract

**Background:**

Although previous studies have identified that both physical activity and sleep problems are independently associated with decreased risk of cognitive function. However, the joint association of physical activity and sleep duration with cognitive function was rarely studied.

**Methods:**

A total of 21,128 participants who had records from the China Family Panel Studies (CFPS) in 2018 were included in this study. Linear regression was used to examine the associations of joint between physical activity and sleep duration with cognitive function in the nationally representative survey data.

**Results:**

Compared with individuals reporting 150 min/week or more of activity, those reporting no physical activity had a 116% higher risk of getting lower vocabulary scores (coefficient: -1.16, 95% CI: −1.55 ~ −0.78) and a 61% higher risk of getting lower mathematics scores (coefficient: -0.61, 95% CI: −0.78 ~ −0.44). Compared with those who slept for 7–10 h/day, those who slept more than 10 h/day had the lower vocabulary scores (coefficient: −1.34, 95% CI: −1.86 ~ −0.83) and mathematics scores (coefficient: −0.68, −0.94 ~ −0.42). The results of joint analysis showed that the adjusted coefficient for vocabulary scores were − 2.58 (95% CI, −3.33 ~ −1.82) for individuals reporting no physical activity and sleeping for 10 h/day, and − 1.00 (95% CI, −1.88 ~ −0.12) for individuals reporting more than 150 min/week and sleeping for 10 h/day, compared with those who reported a sleep duration for 7–10 h/day and more than 150 min/week physical activity, Any level of physical activity combined with longer sleep duration (≥10 h/day) was associated with a higher risk of getting low mathematics scores.

**Conclusion:**

Appropriate sleep and sufficient physical activity together may have amplified association on cognitive performance, highlighting the importance of a comprehensive healthy lifestyle.

## 1. Introduction

Sleep duration, as an important indicator of health, is essential for maintaining an optimal health condition (1). According to the daily sleep duration recommended by the American Academy of Sleep Medicine, both insufficient sleep and longer sleep could increase the adverse health outcomes such as hypertension, diabetes, obesity, and mental health problems (2). Long sleep duration in older adults is associated with morbidity and mortality according to National Sleep Foundation’s updated (3). Both short and long sleep duration on weekdays and weekends are related to depressive symptoms (4). Importantly, developmental differences in sleep may impact cognition and brain development (5), and the underlying mechanism might be the impaired hippocampal neuronal plasticity and memory processes (6).

Exercise modalities have benefits on the health and well-being of adults, improving physical capacities and cognitive function, both in normal states of brain aging and in different stages of cognitive impairment (7). Physical activity was effective in improving global cognition in Alzheimer disease and in all types of dementia (8). Exercise may reduce decline in global cognition in older adults with mild-to-moderate Alzheimer’s disease dementia (9). Physical inactivity and sleep problems are independently associated with decreased risk of cognitive function. Higher total physical activity levels are associated with better sleep quality (10). Although the potential codependency and synergetic effects physical activity and sleep problems have with onset of poor mental health (11), the incidence of hypertension (12) and cardiometabolic health markers (13), few studies examined the association of sleep duration and physical activity with cognitive performance in Chinese population.

Thus, this study firstly evaluates the association of sleep duration and physical activity with cognitive performance including vocabulary and mathematics scores among Chinese population. Subsequently, we assess the joint association of sleep duration and physical activity with cognitive performance.

## 2. Materials and methods

### 2.1. Study design and sample

This study uses data from the China Family Panel Studies 2018 (CFPS) (14), which were funded by the 985 Program of Peking University and carried out by the Institute of Social Science Survey (ISSS) of Peking University. The CFPS is a nationally representative, the annual longitudinal project reviewed and approved by the ISSS of Peking University. Taking into account regional differences and survey costs, CFPS implemented probability proportional to size sampling with implicit stratification. Each subsample in CFPS went through three stages of extraction (districts/counties–villages/communities-households) (14). The 2018 CFPS surveyed about 15,000 households and collected nearly 44,000 copies of questionnaires. The CFPS mainly adopted face-to-face interviews to collect data and 22% were aided by phone interviews.

**Figure 1:**
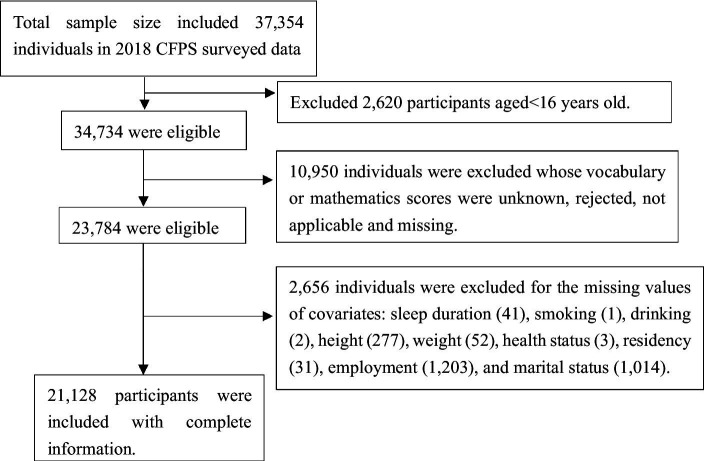
The flow diagram of subject recruitment.

The total sample size included 37,354 individuals. We firstly excluded 2,620 participants aged<16 years old. Subsequently, we excluded the individuals whose index values were unknown, rejected, not applicable and missing: vocabulary and mathematics scores (10,950), physical activity (32), sleep duration (41), smoking (1), drinking (2), self-reported height (277), self-reported weight (52), self-reported health status (3), residency (31), employment (1,203), and marital status (1,014). Finally, our analytic sample included 21,128 participants with complete information. The flow diagram of subject recruitment is shown in Figure 1.

The project was retrieved from the CFPS website for public access to secondary data,^1^ which excludes all identifiable information about individual participants.

This study was conducted in accordance with the Declaration of Helsinki, and all procedures involving human subjects were approved by the Ethics Committee of Peking University (No. IRB00001052–14010). All participants were asked to provide written informed consent before completing the survey.

### 2.2. Assessment of sleep duration and physical activity

In the 2018 CFPS, three items on sleep duration were constructed from relevant survey questions (15), including “On average, how many hours do you sleep each day?,” “On a typical workday, how many hours do you sleep?,” and “On a typical free day, how many hours do you sleep?.” If individuals reported the first question, the latter two questions would be missing; conversely, individuals would report the latter two questions. According to their reply, sleep duration was calculated by (hours on school days*5 + hours on weekends*2)/7 and was categorized as <7 h, 7–10 h, and ≥ 10 h (16).

The duration of physical activity was assessed by the question: “How much time in total do you exercise each week?” (16). Physical activity was subsequently classified as: reporting no activity per week; reporting activity from 1 to 149 min/week; reporting activity ≥150 min/week.

### 2.3. Assessment of cognitive performance

The American psychologist Cattell has divided cognitive ability into two components: fluid intelligence and crystalized intelligence (17). In the CFPS 2018, all individuals performed the cognitive ability tests (including vocabulary and mathematics tests) by self-answer questionnaire (14, 16, 18). The theoretical basis of the CFPS cognitive ability tests was the design of Guttman Scale in psychometry, which showed good reliability and validity (19).

The vocabulary test was consisted of 34 Chinese characters drawn from the language textbooks used in primary and secondary schools and sorted in ascending order of difficulty. This test seeks to measure one’s vocabulary by how difficult he or she can recognize a character and the final score is the rank of the hardest question that the respondent answers correctly. Respondents did not know the rules before taking the test, so they would not fail on purpose. To make the test more efficient, all survey respondents were assigned to one of three entry points, based on their self-reported highest level of education. The respondents were asked to recognize the increasingly difficult characters one by one until they failed to recognize three consecutive characters. The mathematics test was consisted of 24 mathematical questions. The procedures of mathematics test were similar to vocabulary test. The mathematics test scores were categorized based on the same rank-order rule as that in the vocabulary test (18).

### 2.4. Covariates

To provide more compelling evidence by reducing the amount of confounding influence, a set of variables which were previously revealed to associate with cognitive function (i.e., gender, age, educational level, residency, employment, obesity, smoking, drinking, health status, marital status and depression) were adjusted for. Gender, residency status and marital status were defined as dichotomous variables (gender: male or female; residency: rural or urban; marital status: unmarried, married or divorced/widowed/ cohabitation). Age was measured by years, and educational level was measured by asking the respondent to indicate the highest level of education completed: low (completed primary school or lower), middle (completed junior middle school but did not undergo the tertiary entrance exam) and high (took a tertiary entrance exam or higher) (17). The smoking and drinking status were assessed by the question: “Have you used at least one cigarette/drink during the recent month?” The responses were coded as follows: 1 = yes; 2 = no. BMI was calculated through the self-reported height and weight. The obesity was defined as a BMI ≥ 28 kg/m^2^. Health status was measured according to respondents’ self-rated physical health status: good/average and poor. Depressive symptoms were measured by the brief self-report 8-item version of The Center for Epidemiologic Studies Depression Scale (CES-D). The total score of CES-D was from 0 to 24, with a higher score indicating a higher level of depressive symptoms. A cut-off of greater than or equal to 9 has been suggested for this scale as indicating significant depressive symptoms (20).

### 2.5. Statistical analysis

For descriptive purposes, continuous data was grouped into categorical data, and categorical data are presented as numbers and percentages. Linear regression was used to determine the associations among sleep duration, physical activity and cognitive ability. In the model 1, age and sex were adjusted for. In the model 2, education, residency and employment were additionally adjusted for. In the model 3, apart from the variables included in model 2, obesity, smoking, drinking, health status, marital status and depression were additionally adjusted for. In addition, physical activity and sleep duration were adjusted for mutually in the models. Further analysis would be performed to evaluate the association between sleep duration and physical activity with cognitive performance in subgroups of age (16–50 years old or > 50 years old), sex (male or female) and employment (employed or unemployed), respectively. All analyzes were weighted to account for the complex, multi-stage. All analyzes were carried out using STATA 17.0 (Stata Corp, College Station, TX, United States). Statistical significance was set at 0.05.

## 3. Results

The characteristics of each participant are shown in Table 1. Of the 21,128 participants, 51.62% were women, 9.16% were obese, 29.78% smoked. 14.84% participants slept for less than 7 h/day, 65.66% slept for 7–10 h/day and 9.04% slept for 10 h/day. More than half (51.54%) reported no activity, 9.30% reported activity from 1 to 149 min/week, and 39.16% reported activity more than 150 min/week. Participants who had higher degree tend to do more physical activity every week.

**Table 1:** Baseline characteristics of study participants stratified by physical activity and sleep duration in CFPS 2018.

Characteristics	Physical activity (min/week)						Sleep duration (hours/day)					
	0		1–149		> = 150		<7		7- < 10		> = 10	
	*n*	*p* (%)	*n*	*p* (%)	*n*	*p* (%)	*n*	*p* (%)	*n*	*p* (%)	*n*	*p* (%)
Age (years)												
16–50	5,502	56.19	1,148	11.73	3,141	32.08	1,793	18.31	7,278	74.33	720	7.35
>50	5,387	47.52	817	7.21	5,133	45.28	3,552	31	6,594	58.16	1,191	10.51
Sex												
Male	5,776	52.96	1,025	9.4	4,105	37.64	2,879	26.4	7,089	65	938	8.6
Female	5,113	50.02	940	9.2	4,169	40.78	2,466	24.12	6,783	66.36	973	9.52
Education												
Low	6,148	58.52	707	6.73	3,650	34.75	2,891	27.52	6,389	60.82	1,225	11.66
Middle	2,917	49.27	573	9.68	2,430	41.05	1,429	24.14	4,014	67.8	477	8.06
High	1,824	38.78	685	14.57	2,194	46.65	1,025	21.79	3,469	73.76	209	4.44
Urban/Rural												
Urban	4,617	44.84	1,102	10.7	4,578	44.46	2,817	27.36	6,786	65.9	694	6.74
Rural	6,272	57.91	863	7.97	3,696	34.12	2,528	23.34	7,086	65.42	1,217	11.24
Employ												
Yes	8,517	54.48	1,540	9.85	5,577	35.67	3,576	22.87	10,791	69.02	1,267	8.1
No	2,372	43.17	425	7.74	2,697	49.09	1,769	32.2	3,081	56.08	644	11.72
Obesity												
Yes	947	48.94	189	9.77	799	41.29	525	27.13	1,223	63	187	9.66
No	9,942	51.8	1,776	9.25	7,475	38.95	4,820	25.11	12,649	65.9	1,724	8.98
Smoking												
Yes	3,339	53.08	551	8.76	2,401	38.17	1,516	24.1	4,179	66.43	596	9.47
No	7,550	50.89	1,414	9.53	5,873	39.58	3,829	25.81	9,693	65.33	1,315	8.86
Drinking												
Yes	1,848	52.95	297	8.51	1,345	38.46	919	26.33	2,259	64.73	312	8.94
No	9,041	51.26	1,668	9.46	6,929	39.28	4,426	25.09	11,613	65.84	1,599	9.07
Marital status												
Unmarried	726	50.8	218	15.26	485	33.94	207	14	1,100	76.98	122	8.54
Married	9,392	51.84	1,628	8.99	7,097	39.17	4,608	25	11,904	65.71	1,605	8.86
Others	771	48.74	119	7.52	692	43.74	530	34	868	54.87	184	11.63
Self-reported health												
Good/average	8,618	50.44	1,676	9.81	6,793	39.76	4,043	23.66	11,668	68.29	1,376	8.05
Poor	2,271	56.2	289	7.15	1,481	36.65	1,302	32.22	2,204	54.54	535	13.24
Depression												
Yes	10,173	52.15	1,842	9.44	7,494	38.41	5,048	25.88	12,687	65.03	1,774	9.09
No	716	44.22	123	7.6	780	48.18	297	18.34	1,185	73.19	137	8.46

### 3.1. Individual associations of physical activity and sleep duration

Compared with individuals reporting 150 min/week or more of activity, we observed a 116% higher risk of getting lower vocabulary scores (coefficient: -1.16, 95% CI: −1.55 ~ −0.78) and a 61% higher risk of getting lower mathematics scores (coefficient: −0.61, 95% CI: −0.78 ~ −0.44) among those reporting no physical activity. Compared with those who slept for 7–10 h/day, the adjusted coefficient for vocabulary score was −1.34 (95%CI, −1.86 ~ −0.83), and the coefficient for mathematics scores was −0.68 (95% CI, −0.94 ~ −0.42) for individuals reporting sleeping more than 10 h/ day in fully adjusted model. All the results were detailed in Tables 2, 3.

**Table 2:** Association between physical activity with cognitive performance in cross-sectional study.

Cognitive performance	Physical activity (min/week)	Model 1				Model 2				Model 3a			
		Coefficient	95% CI		*p* value	Coefficient	95% CI		*p* value	Coefficient	95% CI		*p* value
Vocabulary scores	0	−3.52	−4.23	−2.81	<0.001	−1.29	−1.68	−0.89	<0.001	−1.16	−1.55	−0.78	<0.001
	1–149	−0.01	−0.58	0.56	0.98	−0.18	−0.61	0.24	0.39	−0.12	−0.53	0.28	0.55
	> = 150	0 (reference)				0 (reference)				0 (reference)			
Mathematics scores	0	−1.81	−2.14	−1.48	<0.001	−0.67	−0.84	−0.50	<0.001	−0.61	−0.78	−0.44	<0.001
	1–149	0.28	−0.08	0.65	0.13	0.14	−0.15	0.44	0.34	0.17	−0.13	0.46	0.27
	> = 150	0 (reference)				0 (reference)				0 (reference)			

Model 1: adjusting for age and sex; Model 2: adjusting for age, sex, education, residency and employment; Model 3a: adjusting for age, sex, education, residency, employment, obesity, smoking, drinking, sleep duration, self-reported health, marital status and depression.

**Table 3:** Association between sleep duration with cognitive performance in cross-sectional study.

Cognitive performance	Sleep duration (hours/day)	Model 1				Model 2				Model 3b			
		Coefficient	95% CI		*p* value	Coefficient	95% CI		*p* value	Coefficient	95% CI		*p* value
Vocabulary scores	<7	0.25	−0.27	0.77	0.35	0.20	−0.16	0.56	0.28	0.25	−0.09	0.60	0.15
	7- < 10	0 (reference)				0 (reference)				0 (reference)			
	> = 10	−2.98	−3.76	−2.20	<0.001	−1.59	−2.13	−1.05	<0.001	−1.34	−1.86	−0.83	<0.001
Mathematics scores	<7	−0.16	−0.41	0.09	0.21	−0.13	−0.31	0.06	0.18	−0.10	−0.28	0.08	0.27
	7- < 10	0 (reference)				0 (reference)				0 (reference)			
	> = 10	−1.58	−1.96	−1.20	<0.001	−0.77	−1.04	−0.51	<0.001	−0.68	−0.94	−0.42	<0.001

Model 1: adjusting for age and sex; Model 2: adjusting for age, sex, education, residency and employment; Model 3b: adjusting for age, sex, education, residency, employment, obesity, smoking, drinking, physical activity, self-reported health, marital status and depression.

### 3.2. Joint associations of physical activity and sleep duration

The joint associations between physical activity and sleep duration with cognitive performance are presented in Table 4. Compared with those who slept for 7–10 h/day reporting more than 150 min/week, the adjusted coefficient for vocabulary scores were −2.58 (95% CI, −3.33 ~ −1.82) for reporting no physical activity with a sleep duration≥10 h/day, and −1.00 (95% CI, −1.88 ~ −0.12) for reporting more than 150 min/week with a sleep duration≥10 h/day. Similarly, those who reporting no physical activity with a sleep duration≥10 h/day had a 130% higher risk of getting lower mathematics scores (95% CI: −1.67 ~ −0.93), 82% for reporting 1 to 149 min/week with a sleep duration≥10 h/day (95% CI: −1.58 ~ −0.05), 53% for reporting more than 150 min/week with a sleep duration≥10 h/day (95% CI, −0.96 ~ −0.09), respectively. Those shorter sleepers (<7 h/day) reporting no activity had lower vocabulary scores (coefficient: -0.83, 95% CI: −1.34 ~ −0.31) and mathematics scores (coefficient: -0.69, 95% CI: −0.95 ~ −0.43).

**Table 4:** Joint association of physical activity and sleep duration with cognitive performance.

Cognitive performance	Physical activity (min/week)	Sleep duration (hours/day)											
		<7				7- < 10				> = 10			
		Coefficient	95% CI		*p* value	Coefficient	95% CI		*p* value	Coefficient	95% CI		*p* value
Vocabulary scores	0	−0.83	−1.34	−0.31	0.002	−1.12	−1.56	−0.69	<0.001	−2.58	−3.33	−1.82	<0.001
	1–149	−0.22	−1.12	0.68	0.63	0.06	−0.44	0.55	0.82	−1.67	−3.52	0.18	0.08
	> = 150	0.32	−0.23	0.88	0.26	0 (reference)				−1.00	−1.88	−0.12	0.03
Mathematics scores	0	−0.69	−0.95	−0.43	<0.001	−0.59	−0.78	−0.39	<0.001	−1.30	−1.67	−0.93	<0.001
	1–149	0.00	−0.48	0.48	1.00	0.24	−0.13	0.61	0.20	−0.82	−1.58	−0.05	0.04
	> = 150	−0.07	−0.36	0.22	0.63	0 (reference)				−0.53	−0.96	−0.09	0.02

Model 4: adjusting for age, sex, education, residency, employment, obesity, smoking, drinking, self-reported health, marital status and depression.

### 3.3. Subgroup analyzes

In the subgroups of age, sex and employment, compared with those reporting a sleep duration for 7-10 h/day, short sleeper (<7 h/day) who are younger than 50 years old had a 35% higher risk of getting lower mathematics scores (coefficient: −0.35, 95% CI = −0.64 ~ −0.07). Those individuals who were employed with a sleep duration ≥10 h/day tend to get lower vocabulary scores (coefficient: −1.47, 95% CI: −2.03 ~ −0.91) and mathematics scores (coefficient: −0.81, 95% CI: −1.11 ~ −0.51). And other results observed were consistent with our primary findings. All the results were detailed in Table 5.

**Table 5:** Subgroups analysis of association between sleep duration and physical activity with cognitive performance.

Cognitive performance	Subgroups	Physical activity (min/week) Model 3a									Sleep duration (hours/day) Model 3b								
		0				1–149				> = 150	<7				7- < 10	> = 10			
		Coefficient	95%CI		*p* value	Coefficient		95%CI	*p* value		Coefficient	95%CI		*p* value		Coefficient	95%CI		*p* value
Vocabulary scores	Age < =50	−0.54	−0.94	−0.15	0.008	0.22	−0.29	0.73	0.40	0 (reference)	−0.33	−0.82	0.16	0.19	0 (reference)	−1.54	−2.28	−0.81	<0.001
	Age > 50	−1.56	−2.13	−0.99	<0.001	−0.20	−1.04	0.65	0.65	0 (reference)	0.68	0.21	1.14	0.004	0 (reference)	−1.37	−2.05	−0.70	<0.001
	Male	−1.44	−1.90	−0.96	<0.001	−0.26	−0.86	0.34	0.40	0 (reference)	0.41	−0.14	0.95	0.15	0 (reference)	−1.19	−1.90	−0.48	0.001
	Female	−0.89	−1.41	−0.37	0.001	0.01	−0.57	0.60	0.97	0 (reference)	0.28	−0.21	0.76	0.27	0 (reference)	−1.73	−2.51	−0.96	<0.001
	Unemployed	−1.73	−2.46	−0.99	<0.001	0.10	−0.97	1.18	0.82	0 (reference)	0.68	0.00	1.35	0.05	0 (reference)	−0.89	−1.95	0.17	0.1
	Employed	−0.94	−1.32	−0.55	<0.001	−0.10	−0.53	0.33	0.65	0 (reference)	0.11	−0.28	0.51	0.51	0 (reference)	−1.47	−2.03	−0.91	<0.001
Mathematics scores	Age < =50	−0.49	−0.73	−0.26	<0.001	0.31	−0.10	0.71	0.13	0 (reference)	−0.35	−0.64	−0.07	0.02	0 (reference)	−0.97	−1.42	−0.52	<0.001
	Age > 50	−0.67	−0.86	−0.47	<0.001	−0.06	−0.48	0.37	0.79	0 (reference)	0.15	−0.07	0.36	0.18	0 (reference)	−0.53	−0.78	−0.27	<0.001
	Male	−0.64	−0.85	−0.44	<0.001	0.23	−0.15	0.60	0.23	0 (reference)	−0.05	−0.30	0.19	0.67	0 (reference)	−0.58	−0.91	−0.24	0.001
	Female	−0.58	−0.82	−0.33	<0.001	0.08	−0.31	0.47	0.69	0 (reference)	−0.11	−0.40	0.17	0.43	0 (reference)	−0.84	−1.23	−0.45	<0.001
	Unemployed	−0.87	−1.16	−0.58	<0.001	−0.13	−0.76	0.50	0.69	0 (reference)	−0.18	−0.49	0.13	0.25	0 (reference)	−0.41	−0.87	0.04	0.07
	Employed	−0.53	−0.72	−0.33	<0.001	0.26	−0.05	0.57	0.11	0 (reference)	−0.07	−0.28	0.15	0.53	0 (reference)	−0.81	−1.11	−0.51	<0.001

Model 3a: adjusting for age, sex, education, residency, employment, obesity, smoking, drinking, sleep duration, self-reported health, marital status and depression.

Model 3b: adjusting for age, sex, education, residency, employment, obesity, smoking, drinking, physical activity, self-reported health, marital status and depression.

## 4. Discussion

In the present study, compared with individuals reporting activity more than 150 min/week, individuals reporting no physical activity had higher risk of getting lower cognitive performance. Participants who reported a sleep duration ≥10 h/day were at higher risk of getting lower scores when compared with those reporting a sleep duration for 7-10 h/day. In the joint associations analysis, compared with those who reporting a sleep duration for 7–10 h/day with doing activity more than 150 min/week, any level of physical activity combined with longer sleep duration was associated with a higher risk of getting lower mathematics scores.

Cognitive impairment is associated with increased risk of disability, increased health expenditures, and progression to dementia problems in memory (21). Evidence showed that lifestyle factors (such as physical, sleep problems, diet, smoking, alcohol drinking) might affect cognitive function (22–26). Both longer and short sleep duration were associated with higher risks of cognitive impairment (27). Doing exercise have positive effect on improving cognitive function (8, 28). An umbrella review evaluated 28,205 participants with mild cognitive impairment or dementia, physical activity was not only effective in improving global cognition in Alzheimer disease and in all types of dementia, but also improved noncognitive outcomes in people with dementia including falls, and neuropsychiatric symptoms (8). In our study, we observed a higher risk of getting lower scores among those reporting no physical activity, comparing with individuals reporting 150 min/week or more of activity. Individuals with a longer sleep duration had a higher risk of getting lower scores when compared with those who slept for 7–10 h/day. Those results indicated that physical inactivity and long sleep duration impaired the cognitive performance respectively, which was consistent with previous studies. Interestingly, those individuals who were employed with a longer sleep duration tend to get lower cognitive performance in the subgroups analysis. One possible explanation for the difference is that the questions answered by individuals unemployed are different from the questions answered by individuals employed, and another likely reason is that there is a difference between the two subgroups. Thus, further research could be designed to explore it in the future.

Previous studies indicated that physical activity is beneficial in improving global cognitive function (8, 28). An Updated Umbrella Review of the 2018 Physical Activity Guidelines Advisory Committee Report suggested that physical activity improved sleep in adults with insomnia symptoms or obstructive sleep apnea (29). However, multi-behavior interventions may leverage the overlap in the potential biological and psychological mechanisms linking physical inactivity and longer sleep duration to cognitive impairment (29). Many previous studies examined the joint association of physical activity and sleep problems with onset of poor mental health (11), the incidence of hypertension (12) and cardiometabolic health markers (13), to our knowledge, our study firstly reported associations between joint categories of physical activity, sleep duration and the cognitive performance. In the joint associations analysis, we found that the effect of physical inactivity combined with long sleep duration was greater than these individual risk factors on the cognitive performance. Thus, our results might give more evidence to clinical physicians or health care providers, promoting a comprehensive healthy lifestyle.

The primary strength of this study is that CFPS is a large national social tracking project with comprehensive coverage and strong representativeness. We assessed physical activity and sleep duration with cognitive performance firstly. However, several limitations should be noted. Firstly, we acknowledged that the information on other variables (e.g., dietary patterns, internet use, insomnia caused by some psychological factors) might serve as important drivers of cognitive ability (17, 30). Health status and level of education were all self-reported, there was no way to actually assess if somebody has omitted important details about their health, this could have affected cognitive performance. Secondly, both physical activity and sleep duration were self-reported, which means it is subject to recall bias, participants may have been misclassified based on their reported level. Thirdly, this cross-sectional design limits the ability to establish a causal relationship, thus more longitudinal and intervention studies with larger sample size are warranted to on the explore the physical activity and sleep duration on cognitive performance. Despite that, our findings could provide valuable information to help develop public health messages and interventions.

## 5. Conclusion

Our study suggested that sleep duration and physical activity together may have amplified association on cognitive performance compared to their independent associations. Clinically, physicians should be aware of this increased risk among longer sleepers who never exercised. Health care providers should ensure that recommendation to promote physical activity and appropriate sleep time are addressed as daily habits, promoting a comprehensive healthy lifestyle.

## Data availability statement

The original contributions presented in the study are included in the article/supplementary material, further inquiries can be directed to the corresponding author.

## Author contributions

HT: conceptualization, data collection, data analysis, and original draft. TW and Y-QJ: review and comment to manuscript. All authors contributed to the article and approved the submitted version.

## Footnotes

^1^https://www.isss.pku.edu.cn/cfps/index.htm
